# Method validation and antioxidant activities of *Hyperacanthus amoenus* and *Carissa bispinosa*

**DOI:** 10.4102/ojvr.v91i1.2182

**Published:** 2024-12-13

**Authors:** Kedibone G. Kgosana, Tirelo Matlala

**Affiliations:** 1 Department of Pharmacology and Therapeutics, Faculty of Medicine, Sefako Makgatho Health Sciences University, Pretoria, South Africa; 2 Department of Chemistry, Faculty of Science and Technology, Sefako Makgatho Health Sciences University, Pretoria, South Africa

**Keywords:** feed additive, nutritional browse, plant extracts, foliage, phenolic acid, high performance liquid chromatography, free radical, antioxidants

## Abstract

Plant foliages used as feed additives pose a health risk due to high oxidant concentrations. Oxidants cause oxidative stress and high rate of morbidities and mortalities. Hence, the aim of the study was to validate the methods to quantify gallic acid (GA) and quercetin (Q) as putative antioxidants, and to evaluate antioxidant activities in feed (F), *Hyperacanthus amoenus* (HA) and *Carissa bispinosa* (CB) extracts. Extraction was carried out with 62.5% methanol. Method validations for linearity, accuracy and precision were performed on high performance liquid chromatography. Quantitative analysis of GA and Q and testing of 2,2-diphenyl-2-picrylhydrazyl (DPPH) scavenging activities in the extracts were performed. The lowest limit of detection (LOD) and limit of quantitation (LOQ) of 0.011 µg/mL and 0.032 µg/mL were determined in HA, respectively. The methods were accurate and precise as the relative standard deviations (%RSD) were less than 15%. The GA concentrations in CB and HA extracts were statistically significant (*p* < 0.05) and their values were 0.65 ± 0.03 x 10_6_ µg/kg dry weight (DW) (0.13%) and 0.28 ± 0.06 x 10_6_ µg/kg DW (0.002%), respectively. All extracts showed very strong radical scavenging activities with their IC_50_ values ranging between 5.87 µg/mL and 6.86 µg/mL.

**Contribution:**

These accurate, repeatable, precise and reliable methods can be used to provide a valuable basis for GA and Q analysis in various shrub foliages. Though high GA concentrations have potential to act as antioxidants, they may have adverse health and growth performance effects when used as feed additives, while lower Q concentrations may have no effects on livestock.

## Introduction

Because of the increasing industry standards and the growing demand for natural supplements in livestock nutrition, the feed industries have pressure in finding eco-friendly and safe substances to improve livestock nutrition (Christaki et al. 2020; Pandey, Kumar & Saxena 2019). Owing to high input costs, toxic residues in feed and resistance associated with the use of antibiotics as feed supplements (Dutta, Yadav & Chatterjee 2019; Kuralkar & Kuralkar 2021), plants have capabilities to improve livestock health and immune functions as they possess antioxidants, antimicrobial and anti-inflammatory activities (Abd-Alla 2022). Thus, it is important to observe that feed and feed ingredients should meet minimum safety standards to guarantee suitability, acceptance, safety and good quality which is of paramount importance to animal production.

Generally, feed additives are intentionally added as substances, microorganisms or preparations in micro-quantities in animal feed to enhance nutrient utilisation, growth, health parameters, production and performance of the livestock (Habtamu et al. 2022; Silveira, Roque-Borda & Vicente 2021). According to Bampidis et al. (2019), feed additives may be a well characterised chemical or agent such as a crystallised amino acid; a mixture of active chemicals or agents each of which is qualitatively and quantitatively defined; or a complex mixture such as plant extracts with various bioactive compounds in which not all constituents can be identified. Hence, they can be classified into two categories: nutrient feed additives and non-nutrient feed additives (Kiran & Deswal 2020). As the name implies, the former can be used for the purpose of nutrition enhancement and includes, but is not limited to, amino acids, minerals and vitamins. While the latter can be used for various health purposes such as increasing feed palatability and growth performance improvement (Pandey et al. 2019). Some examples of non-nutrient feed additives include probiotics, prebiotics, hormones and enzymes.

An environmental risk assessment of complex mixtures such as plant extracts has not been developed as yet to give guidance on how to conduct and report studies concerning the assessment of the safety of feed additives for the environment (Bampidis et al. 2019).

Meanwhile research interventions are poised to guard against inappropriate use of plant extracts as feed additives and to evaluate safety level of nutritional browse. Browse is widely vulnerable to adverse environmental conditions which often trigger generation of reactive oxygen species (ROS) and reactive nitrogen species (RNS) as part of defence mechanism. Although these species, often referred to as oxidants, are produced as by-products of the body’s normal metabolism, and as part of the immune system’s protective activity against invading microorganisms, their abundance may be detrimental as they can result in substantial damage to the cells (Surai et al. 2019).

A balance between production of oxidants (oxidation) and the defence mechanism of antioxidants (reduction) commonly known as redox reaction, must be maintained for physiological processes to function at optimum levels. Therefore, an imbalance that may occur because of an excess production of ROS and RNS and/or deficiency/improper functioning of the antioxidant system leads to oxidative stress (Sorelle, Ferdinard & Narcisse 2019). Oxidative stress plays a pivotal role in several pathological processes and diseases such as sepsis, mastitis, acidosis, ketosis, enteritis, and pneumonia, respiratory, joint diseases and other diseases arising from extreme environmental conditions, which may have serious impact on animal production (Laliotis et al. 2020; Nikolova et al. 2022; Singh et al. 2019). Hence, this study opted to evaluate the safety of *Hyperacanthus amoenus* (HA) (Sims) Bridson and *Carissa bispinosa* (CB) (L.) Desf. ex Brenan leaves that are commonly used as nutritional browse, particularly during a period of feed scarcity. According to South African National Biodiversity Institute Both (n.d.a and n.d.b), these evergreen shrubs grow despite the unfavourable environmental conditions such as drought and wind. Although the fruits of HA have been used for snacking (Welcome & Van Wyk 2019), the antioxidant properties of the entire plant species have not been clearly addressed. Conversely, the fruits of CB were reported to have antioxidant properties (Gwatidzo, Dzomba & Mangena 2018), but not much is known about the safety of its foliage. Henceforth, phenolic compounds such as gallic acid (GA) and quercetin (Q) with antioxidant properties were extracted, quantitatively analysed and compared with the commercial feed to rule out safety doubts and to confirm their potential use as antioxidants.

## Research methods and design

### Plant collection

Fresh leaves of CB and HA were collected during the month of August in 2017 at the Grow Wild Purposefully Indigenous garden in Midrand (-25.98820, 28.04372) in Gauteng province, South Africa. The voucher specimens were deposited in the H.G.W.J Schweickerdt Herbarium of the University of Pretoria and the University of Pretoria herbarium (PRU) numbers 123 736 and 123 738 were designated for CB and HA, respectively.

### Reagents

All analytical grade reagents and standards used in this study were purchased from Sigma-Aldrich (South Africa) and Merck (South Africa), while high performance liquid chromatography (HPLC) grade solvents were purchased from Merck. Bovine feed (F) pellets (code R1153P) (AFGRI, Bethlehem), constituting crude protein, crude fibre, moisture, crude fat, calcium and phosphorus, were used as a negative control for quantitative analysis.

### Processing

The plant collected leaves were washed with tap water, dried under a fan at room temperature for 21 days and ground into powder with a rotor mill, ZM 200 (Retshch GmbH). Similarly, F pellets were also ground into powder.

### Extraction of phenolic compounds

The extracts were prepared using a previously modified method (Proestos & Komaitis 2013). All ground powders were weighed separately and added at a ratio of 1:80 weight/volume into 62.5% methanol in 250 mL sterile bottles. One volume of 6 mol/L HCl was added to each mixture. The mixtures were shaken vigorously for 16 h at room temperature on a shaker (Labotec, model no: 202). The extracts were bubbled for 60 s with nitrogen gas, sonicated for 15 min and heated in a waterbath at 90 °C for 2 h; then, they were filtered through Whatman filter paper number 50 (24.0 cm) and the filtrates concentrated under reduced pressure.

### Quantitative analysis of phenolic compounds

#### Instrumentation, chromatographic conditions and sample preparation

The purpose of this analysis was to quantify phenolic compounds in plant leaves and feed. High performance liquid chromatography analysis was performed using an Agilent 1260 Infinity Quaternary LC (Agilent Technologies). The HPLC instrument comprised of a binary pump, high performance degasser, high performance auto-sampler, column thermostat and a variable wavelength detector. The analytical column used was a Luna^®^ 5 µm C18(2) 100 Å, LC Column 250 mm × 4.6 mm. The chromatographic conditions consisted of 70:30 (methanol: 2% acetic acid in water), 1 mL/min flow rate for 10 min at wavelength detections of 369 nanometre (nm) (Q) and 272 (GA), ambient temperature and injection volumes of 10 µL (Q) and 20 µL (GA). Concentrations of 1 mg/mL stock solutions of GA and Q standards were prepared separately, and appropriate volumes from the stock solution were further diluted to prepare dilute standards of varying concentrations.

#### Method validation

The quantification method was validated for linearity, accuracy, precision, limit of detection (LOD) and limit of quantitation (LOQ) using the following procedures:

#### Linearity

Ten different concentrations ranging from 10 µg/mL to 100 µg/mL were prepared from 1 mg/mL stock solutions of GA and Q. Ten microlitre of Q and 20 µL of GA or Q solutions were injected into the HPLC using auto-sampler, and three replicates were carried out. The calibration curve was generated by plotting the average peak areas against the concentrations using Microsoft Excel 2016. The calibration curve was used to evaluate linearity of the method by calculating the coefficient of correlation, slope and intercept values.

#### Accuracy and recovery

Recoveries of GA and Q were calculated to determine the accuracy of the method. Three known concentrations of standard solutions were added to the analysed sample solutions, and the injections were performed in triplicates. The calibration curve was used to estimate the amount of the standard that was fortified.

#### Precision

Precision was assessed with respect to inter-day, within-laboratory reproducibility (intermediate precision) and repeatability. The inter-day determination was performed over three consecutive days. For repeatability, nine precisions under the same operating conditions over a short interval of time covering the specified range for the procedure (three concentrations/three replicates each) were determined. Within-laboratory reproducibility was determined using two data sets generated by two analysts using the same instrument at different times, and the percent relative standard deviation (RSD) was calculated as intermediate precision.

### Limit of detection and limit of quantification

The LOD and LOQ were determined using the formula:


kσ/S, [Eqn 1]

where *k* is constant (3.3 for LOD and 10.0 for LOQ), σ is the standard deviation of the sample response (blank), and *S* is the slope of the calibration curve.

### 2,2-diphenyl-1-picrylhydrazyl radical scavenging assay

The 2,2-diphenyl-2-picrylhydrazyl (DPPH) radical scavenging activity of feed and plant extracts was determined using a slight modification method proposed by Chaves, Santiago and Alias (2020). A methanolic dilution of DPPH at 10^-4^ molarity was prepared. Aliquots of 1 mL of each sample in methanol were collected (at five different concentrations: 10 µg/mL – 50 µg/mL; two replicates per sample and concentration) and 2 mL of methanolic dilution of DPPH was added. Four concentrations (0.63 µg/mL, 1.25 µg/mL, 2.5 µg/mL and 5 µg/mL) of the standards (GA and Q) were also prepared and treated the same as the samples. The mixture was kept in the dark at room temperature for 16 min, an absorbance was measured at 517 nm using a microplate reader (Biotek uQuant spectrophotometer, Analytical & Diagnostic Products, South Africa). Methanol was used as the blank. The DPPH radical scavenging ability of each preparation was calculated using the following equation:


$$\%\text{ DPPH radical scavenging activity}=\frac{\left({\text{Abs}}_{\left(\text{control}\right)}-{\text{Abs}}_{\left(\text{sample}\right)}\right)}{\left({\text{Abs}}_{\left(\text{control}\right)}\right)}\times 100$$ [Eqn 2]

where Abs_(control)_ is the absorbance of DPPH radical + methanol; Abs_(sample)_ is the absorbance of DPPH radical + sample extract / standard.

### Data and statistical analysis

All measurements for quantitative analysis were carried out in triplicate, and the results were presented as means and standard deviations.

High performance liquid chromatography data were used to construct calibration graphs by plotting mean areas in milli-absorbance units (mAU) against the respective concentrations in µg/mL on Microsoft Excel 2016. While an online Mann–Whitney U test was used as a statistical tool to prove the hypothesis that livestock feeding directly on the leaves of CB and HA acquire high concentrations of GA and Q. While the null hypothesis was that livestock feeding directly on these leaves acquire the same or low phenolic compounds. The *p*-value was calculated, and the significance level was set at 0.05. When the *p*-value was less than the significance level (*p* < 0.05), we rejected the null hypothesis, and the result was considered statistically significant.

### Ethical considerations

Ethical clearance to conduct this study was obtained from the Sefako Makgatho University Research Ethics Committee (SMUREC) (No. SMUREC/S/355/2022:PG).

## Results

### Linearity and sensitivity

Phenolic compounds of bovine F, CB and HA were quantitatively analysed, and their respective methods were validated on HPLC. Various calibration plots showing regression equations with correlation coefficients were drawn and the results were not shown. However, the calibration plots exhibited good linearity over the range tested, with correlation coefficients (*R*^2^) between 0.9906 and 0.999. In terms of the limit of detection and quantitation, the LOQ values of GA in F, CB and HA extracts were 0.25 µg/mL, 2.39 µg/mL and 0.032 µg/mL, respectively, whereas LOD values were 0.083 µg/mL, 0.79 µg/mL and 0.011 µg/mL, respectively. The LOQ values of Q in F, CB and HA were 1.29 µg/mL, 0.58 µg/mL and 0.29 µg/mL, respectively, whereas LOD values were 0.43 µg/mL, 0.19 µg/mL and 0.095 µg/mL, respectively.

### Retention times of quercetin and gallic acid

**FIGURE 1:**
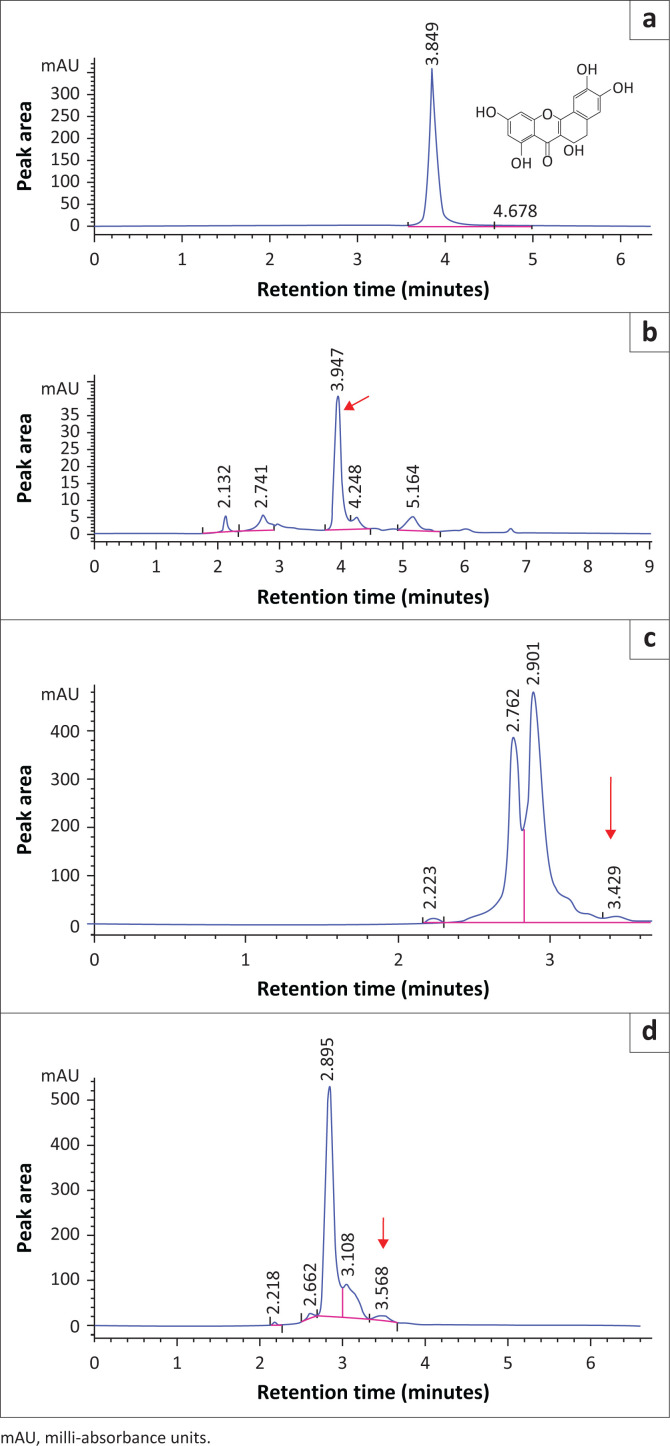
High performance liquid chromatography chromatograms of quercetin (Q) in the mobile phase, feed (F), *Carissa bispinosa* (CB) and *Hyperacanthus amoenus* (HA) blank extracts. (a) Mobile phase; (b) F extract; (c) HA extract; (d) CB extract. The red arrow points at Q.

Four chromatograms of Q and all extracts are shown in Figure 1. The retention time of Q standard was observed at 3.849 min as a sharp peak. The retention times of Q in F, HA and CB extracts were observed at 3.947 min, 3.429 min and 3.568 min, respectively. The peak signal of Q in F extract appeared slightly sharp and intense. However, those of the plant extracts seemed suppressed and broadened, which could raise an issue of matrix effect.

**FIGURE 2:**
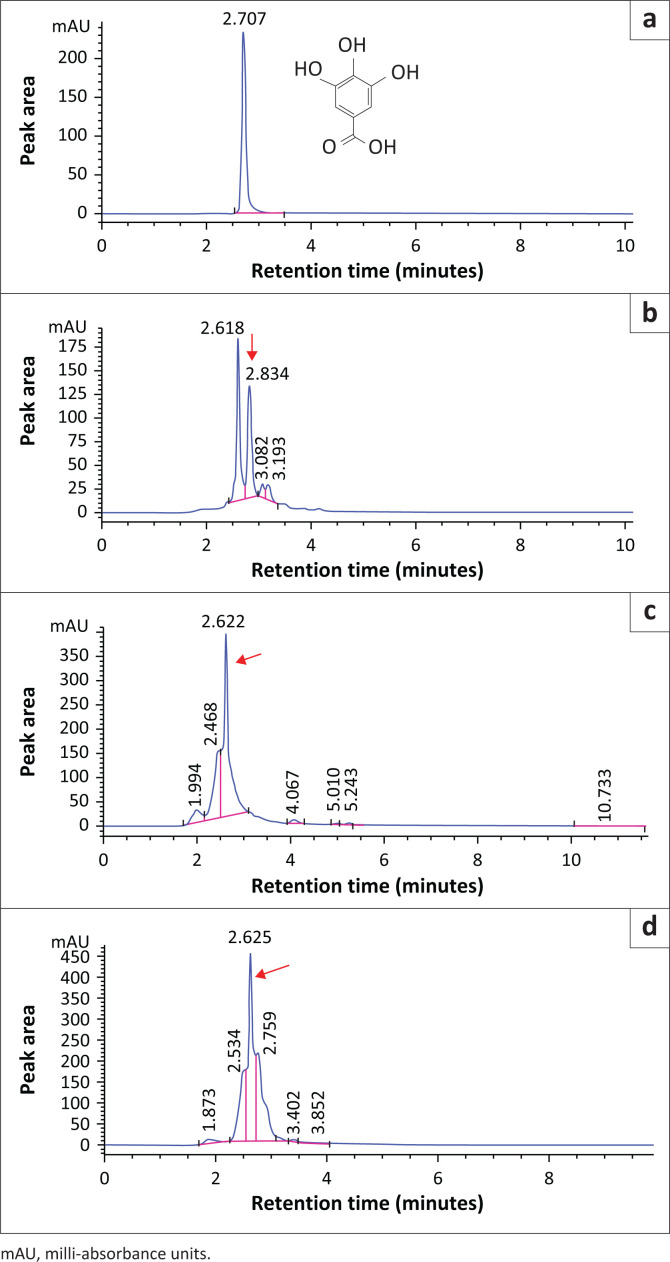
High performance liquid chromatography chromatograms of gallic acid (GA) in the mobile phase, feed (F), *Carissa bispinosa* (CB) and *Hyperacanthus amoenus* (HA) blank extracts. (a) Mobile phase; (b) F extract; (c) CB extract; (d) HA extract. The red arrow points at GA.

Additionally, Figure 2 lists chromatograms of GA and all the extracts. In the mobile phase, GA peak was observed at a retention time of 2.707 min. While in F, CB and HA blank extracts, the peaks were observed at 2.834, 2.622 and 2.625 retention times, respectively.

Again, the GA standard appeared as a sharp and intense peak. Conversely, GA peaks for all the extracts were slightly broadened at the bottom, but sharp at the top. The broadened portions in CB and HA appeared as though the peaks were either splitting or experiencing coelutions.

### Accuracy (recovery) and precision (repeatability)

Three replicates of the selected fortification levels were made to perform recovery of the phenolic compounds, namely, Q and GA (Table 1). In order to confirm the accuracy of the method, the percentage recoveries of Q in F, CB and HA extracts at the selected fortifications ranged between 82.4% – 92.6%, 84.6% – 106% and 100% – 129%, respectively. While the recoveries of GA in F, CB and HA extracts ranged between 91% – 104%, 98.5% – 100%, and 95.8% – 101%, respectively. In addition, the calculated RSD for the Q recoveries in F, CB and HA extracts ranged between 0.10% – 1.19%, 0.17% – 10.7% and 0.30% – 0.69%, respectively. While for GA recoveries in F, CB and HA extracts ranged between 0.94% – 7.25%, 0.13% – 4.75% and 0.24% – 1.04%, respectively.

**TABLE 1:** Recovery and repeatability of gallic acid and quercetin in the extracts.

Sample	Phenolic compound	Spike amount [µg]	Determined concentration [µg] (*n* = 3)	Amount recovered [µg]	% Recovery	% RSD
F	Q	0	3.91	-	-	-
		3	6.24	2.33	90.2	1.19
		6	9.18	5.27	92.6	0.10
		9	10.6	6.73	82.4	0.0
	GA	0	0.076	-	-	-
		2	2.15	2.07	104	7.25
		4	3.78	3.70	92.7	1.18
		6	5.53	5.45	91.0	0.94
CB	Q	0	1.18	-	-	-
		3	4.42	3.24	106	10.7
		6	6.49	5.31	90.4	0.23
		9	8.61	7.43	84.6	0.17
	GA	0	4.48	-	-	-
		2	6.38	1.9	98.5	4.71
		4	8.47	3.99	99.9	4.75
		6	10.5	6.01	100	0.13
HA	Q	0	2.86	-	-	-
		0.74	3.59	0.73	100	0.30
		1.48	4.59	1	106	0.69
		2.22	6.55	3.69	129	0.43
	GA	0	19.7	-	-	-
		2	21.9	2.16	101	1.04
		4	22.8	3.11	96.2	0.24
		6	24.6	4.92	95.8	0.24

CB, *Carissa bispinosa* extract; F, feed extract; GA, gallic acid; HA, *Hyperacanthus amoenus* extract; n, number of replicates; Q, quercetin; RSD, relative standard deviation; µg, microgram.

### Reproducibility and inter-day precision

Within-laboratory reproducibility was determined and the results showed a range between 0.805% – 1.92% and 1.75% – 4.79% for GA and Q, respectively (Table 2).

**TABLE 2:** Determination of within-laboratory reproducibility (*n* = 6).

Extract	µ of the determined concentration (µg/mL)		(σ)		Reproducibility	
	GA	Q	GA	Q	GA	Q
F	5.50	6.37	0.044	0.112	0.805	1.75
CB	5.35	5.94	0.084	0.104	1.57	1.76
HA	5.88	6.03	0.113	0.289	1.92	4.79

CB, *Carissa bispinosa* extract; F, feed extract; GA, gallic acid; HA, *Hyperacanthus amoenus* extract; mL, millilitre; n, number of replicates; σ, standard deviation; Q, quercetin; µ, mean; µg, microgram.

With respect to inter-day precision, at least six replications of the selected fortifications were prepared and analysed to determine precision of the methods. The results showed the RSD of less than 15% for all extracts at all fortification levels (Table 3). Apart from Q levels in CB (day 1 and 3 [both at 3 parts per million {ppm} fortification level]) and F (day 2 and 3 [both at 9 ppm fortification level]), the percentage recoveries of the phenolic compounds ranged between 81.5% and 110%.

**TABLE 3:** Determination of inter-day precision.

Phenolic compound	Extract	Spike [µg/mL]	Day 1 (*n* = 6)				Day 2 (*n* = 6)				Day 3 (*n* = 6)			
			Amount determined (µg/mL)	Amount recovered (µg/mL)	% Recovery	% RSD	Amount determined (µg/mL)	Amount recovered (µg/mL)	% Recovery	% RSD	Amount determined (µg/mL)	Amount recovered (µg/mL)	% Recovery	% RSD
GA	F	0	0.56	-	-	-	0.73	-	-	-	0.73	-	-	-
		2	2.56	1.99	99.8	10.30	2.97	2.23	110.0	0.41	2.93	2.19	109.0	0.38
		4	4.32	3.75	93.4	1.05	4.48	3.75	93.8	0.51	4.48	3.75	93.6	1.04
		6	6.06	5.50	91.6	0.93	6.25	5.51	91.9	0.69	6.27	5.54	92.3	2.10
	CB	0	4.48	-	-	-	4.62	-	-	-	7.72	-	-	-
		2	6.06	1.96	97.9	9.65	6.24	1.86	92.9	10.50	10.5	2.16	108.0	8.01
		4	7.95	3.87	96.8	5.66	8.59	3.55	94.8	5.35	12.4	4.22	105.0	2.24
		6	10.5	6.03	101.0	2.52	10.5	5.86	99.4	1.40	14.5	6.32	105.0	1.97
	HA	0	19.7	-	-	-	4.73	-	-	-	4.37	-	-	-
		2	21.4	1.98	98.8	2.43	5.38	1.86	82.5	3.22	5.20	1.63	81.5	3.26
		4	22.8	3.84	96.0	0.35	8.72	3.79	104.0	1.45	8.89	4.24	106.0	1.59
		6	24.6	5.73	95.5	0.37	9.67	5.97	92.3	0.74	9.75	5.64	93.9	3.31
Q	F	0	3.91	-	-	-	3.05	-	-	-	0.00	-	-	-
		3	6.24	2.32	90.2	1.19	5.27	2.21	87.0	1.13	1.64	2.00	62.0	1.56
		6	9.15	5.24	92.3	1.22	7.77	4.72	85.8	1.75	4.69	5.04	83.0	2.50
		9	10.6	6.65	81.8	2.12	9.28	6.22	77.0	2.52	6.40	6.76	74.0	3.76
	CB	0	0.18	-	-	-	1.18	-	-	-	0.73	-	-	-
		3	3.88	3.73	116.0	11.80	4.58	3.41	109.0	10.20	4.38	3.65	117.0	1.02
		6	6.24	6.03	97.6	1.40	6.49	5.31	90.4	0.42	6.05	5.32	91.4	4.33
		9	8.09	7.92	86.3	0.81	8.89	7.43	84.5	0.86	8.49	7.77	87.3	0.82
	HA	0	2.80	-	-	-	2.86	-	-	-	2.42	-	-	-
		0.74	3.79	0.99	107.0	6.20	3.57	0.71	99.2	5.94	3.30	0.89	104.0	7.46
		1.48	4.69	1.89	109.0	2.09	4.57	1.72	105.0	2.97	4.39	1.97	110.0	4.49
		4	6.92	4.12	101.0	0.02	6.64	3.78	96.8	3.16	6.41	3.99	99.9	0.93

CB, *Carissa bispinosa* extract; F, feed extract; GA, gallic acid; HA, *Hyperacanthus amoenus* extract; n, number of replicates; Q, quercetin; mL, millilitre; RSD, relative standard deviation; µg, microgram.

### Quantitative analysis

**FIGURE 3:**
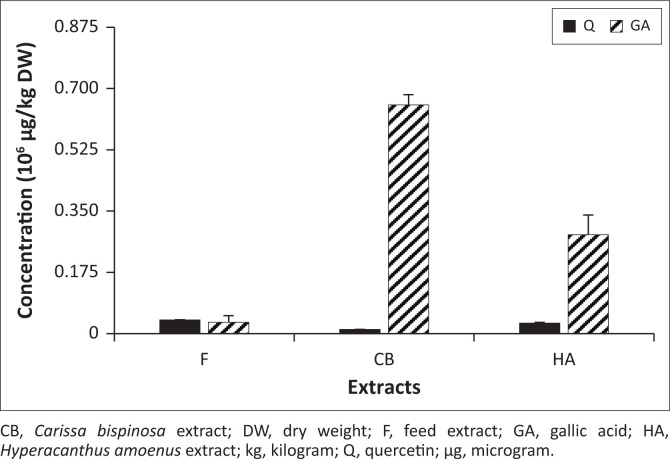
Determined concentrations of phenolic compounds in feed, *Carissa bispinosa* and *Hyperacanthus amoenus* per dry weight. The bars represent mean concentrations in 10^6^ µg/kg dry weight, while error bars represent standard deviations. All *p*-values were less than 0.05 to indicate statistical significance.

Subsequently, the extracts were then analysed to determine the concentrations of the selected phenolic compounds. Figure 3 shows the concentrations of all extracts that ranged between 0.032–0.65 × 10^6^ µg/kg dry weight (DW) and 0.012–0.039 × 10^6^ µg/kg DW for GA and Q, respectively.

The highest concentration in the CB extracts constituted 0.13% GA in the dry plant material (Table 4). While the lowest concentration determined in the F extract constituted 0.002% GA in the dry feed material.

**TABLE 4:** Percentage levels of phenolic compounds (*n* = 6) per dry feed pellets and foliages.

Phenolic compound	Extract	µ	σ
% GA	F	0.0016	0.00081
	CB	0.1257	0.0071
	HA	0.05241	0.00931
% Q	F	0.0038	0.0001
	CB	0.0012	2.3E-05
	HA	0.0029	0.00021

CB, *Carissa bispinosa* extract; F, feed extract; GA, gallic acid; HA, *Hyperacanthus amoenus* extract; n, number of replicates; σ, standard deviation; Q, quercetin; µ, mean.

On the other hand, the determined Q concentrations in the F, CB and HA extracts were 0.039 ± 0.001, 0.012 ± 0.0001 and 0.03 ± 0.002 × 10^6^ µg/kg DW, respectively. The highest concentration in F extract constituted 0.004%. Although this was high relative to HA and CB, the results were statistically significant (*p* < 0.05).

### 2,2-Diphenyl-2-picrylhydrazyl radical scavenging activities

**FIGURE 4:**
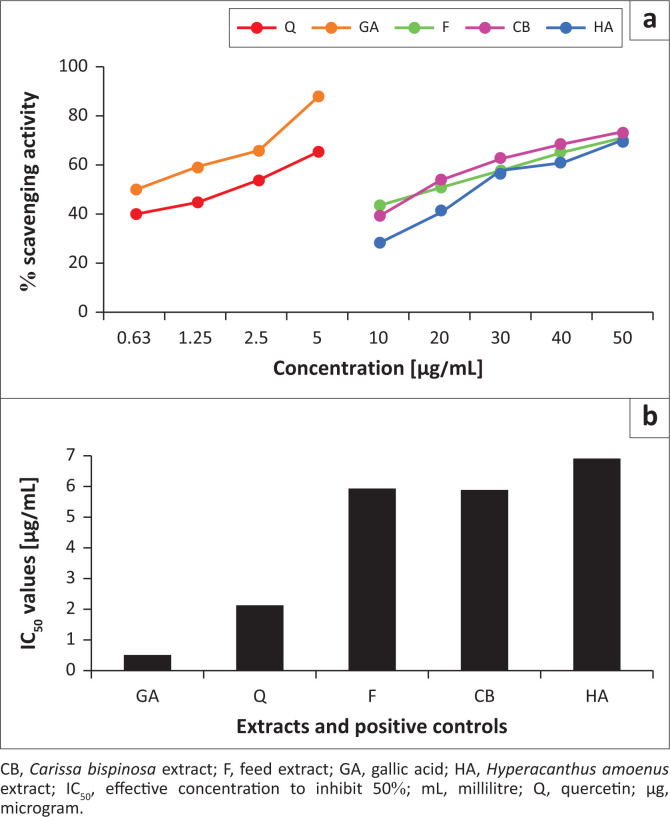
2,2-diphenyl-2-picrylhydrazyl radical scavenging activities and effective concentration to inhibit 50% (IC_50_) values of the extracts and the standards. (a) Percentage scavenging activities. (b) IC_50_ values.

Radical scavenging activities of all extracts and standards were determined and represented in percentages (Figure 4a). They increased with the increasing concentrations. The effective concentration to inhibit 50% (IC_50_) values were determined and they ranged between 0.49 µg/mL and 6.86 µg/mL (Figure 4b).

Nonetheless, the overall potency trend of all samples tested in an increasing order was GA > Q > CB > F > HA. Thus, GA with an IC_50_ value of 0.49 µg/mL was considered the most potent phenolic compound in scavenging the DPPH radicals. On the other hand, HA with an IC_50_ value of 6.86 µg/mL was considered the least potent extract, whereas CB (IC_50_ value = 5.87 µg/mL) was the most potent extract in this study.

## Discussion

The aim of the study was to extract the phenolic compounds found in CB and HA foliages commonly used as feed additives and also as nutritional browse during a feed scarcity period. Unfortunately, the period of feed scarcity is often associated with unfavourable environmental conditions such as drought, UV radiation, high or low temperatures that cause dramatic alterations in the physical state and chemical compositions of plant cells (Rogowska & Szakiel 2020). These stressful conditions frequently lead to overproduction of free radicals, especially the ROS known to cause oxidative cell damage (Rizzo et al. 2007). Therefore, in the event where ROS and/or RNS productions have been increased because of any unfavourable environmental condition leading to the damage of the deoxyribonucleic acid (DNA), proteins, membrane lipids and photosynthetic apparatus, plants have developed defensive mechanisms that involve various enzymes, specialised metabolites and the accumulation of various compounds such as antioxidants to neutralise these species. Hence, method validations were carried out for quantitative analysis of GA and Q in all the extracts. Extracts were also evaluated for their antioxidant properties.

### Linearity and sensitivity

The results for method validations showed good correlation coefficients (*R*^2^), which were greater than 0.99 to indicate contribution of the standard concentrations in predicting the absorbance with more than 99% accuracy. In terms of assessing the sensitivity of analytical methods, the LOQ and LOD were selected as common parameters used to determine the minimum amounts of analytes in a sample that can be quantified and detected, respectively (Ershadi & Shayanfar 2018). It is noteworthy that many studies previously reported lower LOD values than LOQ values regardless of the instrument used for analysis and the nature of the analytes (Abedini et al. 2021; Iqbal & Jeongkwon 2021; Maggira et al. 2021; Onopiuk et al. 2022). Hence, a similar trend was observed in this study. Furthermore, it was not a surprise that the determined LODs and LOQs of GA ranging between 0.011 µg/mL – 2.39 µg/mL and Q (0.095 µg/mL – 1.29 µg/mL) were not comparable with the previous reports as this could be attributed to sensitivity of the analytical instrument and the effects of various components of the extracts. Nonetheless, these low values indicate more sensitivity, suitability and reliability of the analytical method used for analysing these phenolic compounds. Although other studies reported good sensitivity results particularly for analysing these molecules, the LOQ and LOD values were a slightly high as compared to the results obtained in this study. For instance, Thida, Toyama and Satiraphan (2020) reported LOD and LOQ of 0.24 µg/mL and 0.73 µg/mL for GA and 0.21 µg/mL and 0.63 µg/mL for Q in *Madhuca longifolia* (J.Koenig ex L.) Macbr.

### Retention times of quercetin and gallic acid

With regard to the retention times of Q and GA, fortification of various standard concentrations was performed for the purpose of identifying the correct peak as there were a number of peaks around the retention times of these phenolic compounds. Appearance of these peaks could be because of the matrix phenomenon. In chemical analysis, matrices are components of a sample other than the analyte(s) of interests, that often affect the experimental results (Raposo & Barceló 2021). This kind of an influence is referred to as matrix effect and often occurs when molecules coelute with the compound/s of interest (Taylor 2005). Many sensitive analytical instruments including, but not limited to, HPLC are susceptible to the matrix effects. These effects can impede accuracy, sensitivity and reliability of the analytical technique and ultimately present challenges to the analytical process (Williams et al. 2023). The determination of the matrix effects is important when carrying out method validation so as to rule out any doubt. Unfortunately, it was not investigated in the study.

Although the retention times shifted in all blank extracts when Q was analysed, signal suppression and broadening were observed. Thus, signal suppression in CB and HA could be attributed to high mass flows and coelution of compounds such as proteins, lipids, salts, high concentrations of sugar, neutralising charge of the target molecules, accumulation of positively charged ions or other secondary metabolites (Rossmann et al. 2015). Similarly, GA peaks of the blank extracts showed slight shifts of the retention times relative to the GA standard, peak enhancement and broadening, particularly in CB and HA. All these observations could also be associated with the matrix effects. Furthermore, Rossmann et al. (2015) evaluated 33 pharmaceutical molecules and found that strong signal enhancement was because of the presence of at least one hydroxyl-group. Therefore, GA peak enhancement in CB and HA could be as a result of the presence of both free and conjugated forms of GA where the latter has many hydroxyl groups.

### Accuracy (recovery) and precision (repeatability)

Precision with respect to repeatability and accuracy (recovery) were evaluated using recovery tests and adding known amounts of the standards of phenolic compounds to the extracts. The recoveries as determined in terms of RSD were found to be within the acceptable ranges because they were less than 15% RSD, the precision limits for analytical methods, as a function of concentration, which was adopted by Codex Guidelines (n.d.). While average recoveries of 80% – 100% should be obtained when the analytical method can be performed with acceptable precision (Codex Guidelines n.d.). Apart from the analysis of Q in HA at the fortification level of 2.2 mg with 129% recovery, all percentage recovery ranges of the phenolic compounds were found to be within the acceptable range limits (80% – 110%). These results demonstrated the applicability of the developed method for analysis of the phenolic compounds.

### Reproducibility and inter-day precision

In terms of precision that was based on inter-day, the percent recoveries were found to be within the acceptable range, except for the analysis of Q in CB (day 1 and 3) and F (day 2 and 3). The reproducibility (within-laboratory) was also found to be within the acceptable range as less than 5% were obtained. Even so, these results suggested that the methods were precise, reproducible and reliable for analysis of the phenolic compounds.

### Quantitative analysis

Although the lowest GA concentration of 0.032 × 10^6^ µg/kg DW (0.002%) determined in the F extract was above the normal concentration of 0.025 × 10^6^ µg/kg as a proposed dose for feed use (Aquilina et al. 2012), it was at least lower than the proposed dose of 0.125 × 10^6^ µg/kg, which was considered the maximum concentration for safe use in feed. This was expected because a commercial feed has to comply with the safety regulations for feed additives, preservatives and other nutritional supplements. Therefore, it is clear that the feed used in this study underwent rigorous testing and monitoring during the manufacturing processes before deemed acceptable for use and safe for livestock consumption. In contrast, the GA concentrations in the CB (0.65 × 10^6^ µg/kg DW; 0.13%) and HA (0.28 × 10^6^ µg/kg DW; 0.05%) extracts were higher than the proposed dose for feed use. Their use as nutritional browse may raise safety concerns because high levels of GA have anti-nutritive and toxic effects.

High GA concentrations in plants may be attributed to the presence of tannins as GA exists in both free form or conjugated form (gallotannins) (Kim et al. 2021). Such a high concentration in this study was expected as a mere confirmation to the reports from previous studies. For instance, Mashilo, Mashabela and Masoko (2023) reported highest tannin concentration of 13.06 mg gallic acid equivalent (GAE)/g in the hexane extract of CB. Nevertheless, tannins generally exert their anti-nutritive effects as they bind and precipitate dietary proteins to form insoluble complexes that render these proteins inaccessible for metabolism. For instance, Hagerman and Robbins (1993) reported tannins to have a strong affinity to proline-rich proteins in saliva of some animals and the resulting insoluble complex resisted both microbial or ruminant fermentation and enzymatic degradation.

In addition, GA has been reported to have inhibited the growth of *Spodoptera litura* larvae by either exerting its antifeedant or toxic effects (Punia et al. 2021). This was observed with the decrease of ECD (efficiency of conversion of digested food) and ECI (efficiency of conversion of ingested food) with an increase of GA in *S. litura* larvae. In addition, the toxicity of GA to *S. litura* larvae was because of the inhibitory effect on digestion and reduced efficiency of conversion of assimilated food into biomass. However, some livestock can tolerate high GA concentrations in feed for the purpose of health enhancement. This was demonstrated in Mašek, Starčević and Mikulec (2014) where GA supplementation of 5 × 10^6^ µg/kg showed to be beneficial for the fermentation processes in the caecum of broilers.

Furthermore, the endogenous enzymatic antioxidants, commonly referred to as the first-line defence antioxidants, are known to rapidly suppress the formation of free radicals or reactive species in cells (Ighodaro & Akinloye 2018). They include superoxide dismutase (SOD), catalase (CAT) and glutathione peroxidase, and their activities may be significantly enhanced by the presence of phenolic compounds. For instance, the effects of growth, plasma metabolites and bacterial community in the rumen fluid and faeces were evaluated after feeding the preweaning Holstein calves with 0.5 g/kg – 1.0 g/kg of GA (Xu et al. 2022). The results showed a lineal increase of CAT and T-AOC (total antioxidant capacity) as well as a decline in malondialdehyde and tumour necrosis factor-α concentrations. While Owumi et al. (2020) showed the potency of 0.02 g/kg of GA in relieving manganese-induced oxidative stress, lipid peroxidation, and glutathione depletion on hepatorenal function in rats exposed to manganese. Therefore, based on the previous studies, the GA concentrations (0.032 µg/kg – 0.65 µg/kg DW) obtained in this study may indisputably have sufficient potency to enhance CAT and T-AOC as well as reducing oxidative stress in the livestock.

Conversely, Q concentrations determined in this study were relatively low as they ranged between 0.012 and 0.039 × 10^6^ µg/kg DW. The highest concentration was determined in the F extract, which constituted 0.004% in dry feed material. Although there is no prior record regarding the Q concentrations in the plant species investigated in this study, previous research showed that relatively low Q concentrations of 0.0018 and 0.004 × 10^6^ µg/kg were determined in *Hypericum perforatum* foliage and *Sambucus nigra* flowering tops, respectively (Wach, Pyrzyńska & Biesaga 2007). Whereas Q concentrations of 0.281 and 0.094 × 10^6^ µg/kg dry matter were determined in *Moringa oleifera* and *Aloe barbadensis*, respectively (Sultana & Anwar 2008). In order to show health benefits in feed, Zhang and Kim (2020) reported that supplementation of 250 mg/kg – 1000 mg/kg of Q resulted in an increase of body weight gain (BWG) that contributed to growth performance. Interestingly, the maximum response was obtained at 250 mg/kg supplementation. Otherwise, low Q concentrations cannot be expected to have any significant effect on growth performance should both foliages be supplemented in the broilers’ diet.

In terms of endogenous enzymatic antioxidants activity enhancement, Xu et al. (2019) demonstrated the effects of feeding grass carp (*Ctenopharyngodon idella*) on diets supplemented with various concentrations of Q ranging between 0.1 g/kg and 0.8 g/kg. The results showed an increase of SOD and alkaline phosphatase activities with the addition of 0.4 g/kg – 0.6 g/kg of Q (Xu et al. 2019). While another study also showed that increasing dose range from 0 mg Q/kg to 1000 mg Q/kg could linearly increase the content of T-AOC and total SOD (Zhang & Kim 2020). Therefore, the low Q concentrations obtained in the study may have no significant effect on the activities of the endogenous enzymatic antioxidants.

### 2,2-Diphenyl-2-picrylhydrazyl radical scavenging activities

The phenolic extracts were also evaluated for their scavenging activities against DPPH free radicals. As expected, GA and Q standards showed the highest scavenging activities of 90% and 65% at lowest concentrations, respectively. But then, if these activities were classified according to the IC_50_ values as either very strong (< 50 µg/mL), strong (50 µg/mL – 100 µg/mL), medium (101 µg/mL – 150 µg/mL) or weak (> 150 µg/mL) as reported by Fidrianny and AnnisaRuslan (2016), all the extracts and standards would fall under very strong category because their range was 0.49 µg/mL – 6.86 µg/mL. Despite the lack of results for other categories, GA (with IC_50_ value of 0.49 µg/mL) proved to be a stronger DPPH scavenger than Q (IC_50_ value of 2.13 µg/mL). This supported the reports from previous studies, particularly Kim et al. (2013), where GA was shown to have stronger DPPH scavenging activities, with lower IC_50_ value of 19.8 µmol/L than Q (IC_50_ value of 0.7 µmol/L).

### Strengths and limitations

Between-laboratory reproducibility was not conducted because of unavailability of the second analytical instrument or analytical laboratory.

As a result of limited resources and insufficient funding, the study conducted only one radical scavenging activity while there were plenty of methods to select from. However, it would be sensible to explore various scavenging activity methods when resources and funding status change.

### Implications or recommendations

The study focused on analysis of GA, which appears both in a free form and gallotannin form. Therefore, it would be good if further studies could focus on identifying the most dominant form particularly in CB in order to be sure as to whether the nutritional browse could serve as either an anti-nutritive factor, antifeedant, toxin or antioxidant.

## Conclusion

The methods used in this study to determine the GA and Q concentrations in CB and HA foliages were accurate, repeatable, precise and reproducible. They can indisputably provide the basis for quality determination of GA and Q from various shrubs that are often used as feed additives or nutritional browse, particularly during feed scarcity periods. Hence, livestock feeding on CB and HA as nutritional browse acquire high concentrations of GA, which may have adverse effects on general health, nutritional benefits and growth performance due to the abundance of conjugated forms of GA (gallotannins) known to exert their anti-nutritive, antifeedant, and toxic effects. Otherwise, both HA and CB foliages may be useful as good sources of GA with antioxidant properties in the livestock.
